# Supplementary material for: Short-Term Effects of Salt Restriction via Home Dishes Do Not Persist in the Long Term: A Randomized Control Study
Source: Nutrients. 2020 Oct 3;12(10):3034. doi: 10.3390/nu12103034 (PMC7600707; doi:10.3390/nu12103034)
Supplement: Supplementary file 1 [file nutrients-12-03034-s001.pdf]

nutrients

	Coefficient	Sum of squares	df	Mean square	F	P
Monitoring intervention						
Monitoring (ref = None)	125.96	3105679.4	1	3105679.4	1.13	0.29
Seasoning (ref = None)	108.60	2423694.7	1	2423694.7	0.88	0.35
Sex (ref = male)	379.00	5299909.0	1	5299909.0	1.93	0.17
Alcohol drinking habits (ref = None)		4008310.5	2	2004155.3	0.73	0.48
Occasionally	371.25	–	–	–	–	–
Every day	212.04	–	–	–	–	–
Medication status (ref = None)	728.47	13875082.3	1	13875082.3	5.04	0.03
Region (ref = West)	225.07	2232522.0	1	2232522.0	0.81	0.37
Monitoring*Seasoning (Interaction term of the interventions)	253.29	733569.9	1	733569.9	0.27	0.61
REGRESSION	–	28341203.4	8	3542650.4	1.29	0.25
ERROR	–	492571931.8	179	2751798.5	–	–
Seasoning intervention						
Monitoring (ref = None)	272.19	5739948.1	1	5739948.1	3.37	0.07
Seasoning (ref = None)	-202.52	711239.4	1	711239.4	0.42	0.52
Sex (ref = male)	-339.98	3874290.7	1	3874290.7	2.27	0.13
Alcohol drinking habits (ref = None)		2432497.4	2	1216248.7	0.71	0.49
Occasionally	270.23	–	–	–	–	–
Every day	85.25	–	–	–	–	–
Medication status (ref = None)	344.71	3044899.8	1	3044899.8	1.79	0.18
Region (ref = West)	83.95	309182.0	1	309182.0	0.18	0.67
Baseline urinary Na excretion	-0.72	189208235.7	1	189208235.7	111.02	<.0001
Monitoring*Seasoning (Interaction term of the interventions)	157.21	282435.6	1	282435.6	0.17	0.68
REGRESSION	–	217549439.1	9	24172159.9	14.18	<.0001
ERROR	–	303363696.1	178	1704290.4	–	–

Table S1. The summary of ANCOVA at the end of intervention.
